# BAC CGH-array identified specific small-scale genomic imbalances in diploid DMBA-induced rat mammary tumors

**DOI:** 10.1186/1471-2407-12-352

**Published:** 2012-08-15

**Authors:** Emma Samuelson, Sara Karlsson, Karolina Partheen, Staffan Nilsson, Claude Szpirer, Afrouz Behboudi

**Affiliations:** 1 Department of Clinical Genetics, Institute of Biomedicine, Sahlgrenska Academy, University of Gothenburg, SE-40530, Göteborg, Sweden; 2 Department of Oncology, University of Gothenburg, SE-413 45, Göteborg, Sweden; 3 Department of Mathematical Statistics, Chalmers University of Technology, SE-412 96, Göteborg, Sweden; 4 IBMM, Université Libre de Bruxelles, B-6041, Gosselies, Charleroi, Belgium; 5 Systems Biology Research Centre, School of Life Sciences, University of Skövde, SE-54128, Skövde, Sweden

**Keywords:** BAC CGH-array, SPRD-Cu3, DMBA, Mammary tumor, Oncotree model

## Abstract

**Background:**

Development of breast cancer is a multistage process influenced by hormonal and environmental factors as well as by genetic background. The search for genes underlying this malignancy has recently been highly productive, but the etiology behind this complex disease is still not understood. In studies using animal cancer models, heterogeneity of the genetic background and environmental factors is reduced and thus analysis and identification of genetic aberrations in tumors may become easier. To identify chromosomal regions potentially involved in the initiation and progression of mammary cancer, in the present work we subjected a subset of experimental mammary tumors to cytogenetic and molecular genetic analysis.

**Methods:**

Mammary tumors were induced with DMBA (7,12-dimethylbenz[a]anthrazene) in female rats from the susceptible SPRD-Cu3 strain and from crosses and backcrosses between this strain and the resistant WKY strain. We first produced a general overview of chromosomal aberrations in the tumors using conventional kartyotyping (G-banding) and Comparative Genome Hybridization (CGH) analyses. Particular chromosomal changes were then analyzed in more details using an in-house developed BAC (bacterial artificial chromosome) CGH-array platform.

**Results:**

Tumors appeared to be diploid by conventional karyotyping, however several sub-microscopic chromosome gains or losses in the tumor material were identified by BAC CGH-array analysis. An oncogenetic tree analysis based on the BAC CGH-array data suggested gain of rat chromosome (RNO) band 12q11, loss of RNO5q32 or RNO6q21 as the earliest events in the development of these mammary tumors.

**Conclusions:**

Some of the identified changes appear to be more specific for DMBA-induced mammary tumors and some are similar to those previously reported in ACI rat model for estradiol-induced mammary tumors. The later group of changes is more interesting, since they may represent anomalies that involve genes with a critical role in mammary tumor development. Genetic changes identified in this work are at very small scales and thus may provide a more feasible basis for the identification of the target gene(s). Identification of the genes underlying these chromosome changes can provide new insights to the mechanisms of mammary carcinogenesis.

## Background

Genetic aberrations such as deletions and amplifications are known to be involved in tumor initiation and progression [1-3] and their analysis may provide valuable information about regions of the genome harboring cancer-related genes. Today, several techniques are available to detect chromosomal copy number changes and aberrations, namely Karyotyping (G-banding, resolution about 10 megabases, Mb) metaphase based Comparative Genome Hybridization (M-CGH, resolution about 5 Mb) and array-CGH. The BAC (bacterial artificial chromosome) CGH-array technique uses a platform composed of genomic DNA fragments cloned in BAC clones. This method provides a reasonably high resolution (hundreds kilobases, kb) and generates high signal to noise ratios [4,5].

Breast cancer is the most common cancer type affecting women. Development of breast cancer is a multistage process influenced by hormonal and environmental factors as well as by genetic background. The search for genes underlying the disease has recently been highly productive [6,7], but the etiology behind this complex disease is not understood [8]. Several cancer genes have been identified for hereditary breast cancer in human [6], however, these genes account only for a minority, approximately 20%, of genetic risk in hereditary breast cancer [7]. The majority (over 70%) of the hereditary cases are thought to be caused by the interactions of multiple genes with low-penetrance and most of these genes remain to be identified.

In studies using animal cancer models, heterogeneity of the genetic background and environmental factors is reduced and thus analysis and identification of genetic aberrations in tumors may become easier. Genetic component of mammary cancer susceptibility is particularly obvious in the rat models, since different inbred strains, each representing a defined and limited gene pool, exhibit widely different susceptibility to both spontaneous and induced mammary cancer [7,9,10]. There are inbred strains that are almost completely resistant to mammary cancer (e.g. COP, WKY), whereas others are extremely cancer-prone (e.g. WF, SPRD, BUF, ACI). Although it would be quite feasible to analyze the biology behind spontaneously occurring mammary tumors in these model systems, most researchers prefer to use tumor induction with carcinogenic agents in order to reduce latency time and increase incidence of tumor development in the susceptible animals. Commonly used chemical carcinogenic agents for this purpose are NMU (N-methyl-nitrosurea) and DMBA (7,12-dimethylbenz[a]anthrazene). If given in proper dosage at a given time, these chemicals will induce tumors in susceptible animals (such as WF and SPRD) within a few weeks after induction, whereas animals from the resistant strains will not be affected [9,11,12]. Similarly, chronic treatment of ACI rats with estradiol induces a high frequency of mammary tumors, while resistant strains show no tumors in these circumstances [10]. Experimental designs using these models are being used in the search of genes responsible for susceptibility or resistance to developing mammary tumors as valuable tools for genetic studies of breast cancer [7,10].

A major question in cancer genetics is whether certain genomic imbalances are early events and thus the initial force of tumorigenesis [3]. As many cancers have heterogeneous genetic causes, interpretation of data from genetic analysis is found to be difficult. To this end, mathematical methods may aid to determine order of genetic events and thus help to define the pathogenic road(s) taken in the tumor mass [13]. Based on the accepted theory that cancer evolve clonally from a single cell, mathematical analysis may offer guidance for identification of the possible initial deviation in the first cancer cell as representative of starting event during tumorigenesis. This approach might additionally help to determine the evolution of events rooted from this initial deviation resulting in building of the tumor mass [14]. Advanced mathematical data handling algorithms, such as oncogenic tree models, can be used to analyze BAC array data towards identification of important small-scale changes during tumorigenesis [15].

In this study, we subjected a set of 52 DMBA-induced rat mammary tumors to G-banding and M-CGH analyses. Based on the results, 28 of the tumors were selected for a more detailed cytogenetic analysis using an in-house developed BAC CGH-array platform. The result was then used to construct an oncogenetic tree model showing the possible initial events and the development thereof, and the potential order of the observed alterations as well as their correlation to each other during development of these tumors.

## Methods

### Tumor induction with DMBA

SPRD-Cu3 is an inbred rat strain highly prone to develop induced mammary tumors by a single dose of DMBA at a certain age of life. WKY female rats, on the other hand, are resistant to mammary tumor induction with DMBA (described in detail elsewhere, [7,16,17]). SPRD-Cu3 females were mated to WKY/E56 males (hereafter WKY), and F1 males were backcrossed to female SPRD-Cu3 rats. Tumors were induced in female rats from SPRD-Cu3 (n = 10), F1 (n = 32) and SPRD-Cu3x(SPRD-Cu3xWKY) backcross progeny (n = 187) as previously explained [18]. Briefly, at the age of 53–58 days, female rats were given a single gastric dose of 65 mg/kg of DMBA dissolved in sesame oil, per kilogram of body weight. Tumors were detected in the mammary glands by weekly palpations after treatment. Animals were sacrificed and possible tumors were isolated 19 weeks post-treatment or earlier in case of tumor perforating the skin [18]. Tumors were surgically removed from each animal and DNA was extracted. The tumors were subjected to histological analysis and were shown to be papillary adenocarcinomas, sometimes invasive with a cribriform pattern as described earlier [18,19]. A total of 52 mammary tumors, including 11 tumors from SPRD-Cu3, 6 tumors from F1 and 35 tumors from the backcross progeny were included in this study (Table 1). All animal experiments had been approved by the local ethical committee (Université Libre de Bruxelles, Gosselies Belgium).

**Table 1:** Tumor material used in this study

**The genetic background**	**Total number of tumors**	**Number of tumors analyzed by:**		
		**Karyotyping**	**M-CGH**	**BAC CGH-array**
SPRD-Cu3	11	10	11	4
(SPRD-Cu3xWKY)F1	6		6	6
SPRD-Cu3x(SPRD-Cu3xWKY) backcross	35		35	18

Using DMBA, mammary tumors were induced in female rats from three different genetic backgrounds.

### Cytogenetic analysis

To set up primary cell cultures, small pieces of 10 fresh tumor tissues developed in inbred SPRD-CU3 were cultured in Dulbecco´s Modified Eagle Medium (DMEM) containing 10% fetal calf serum supplemented with Glutamine and incubated with standard culture conditions. Primary tumor cell cultures were successfully established for all of the tumors. Chromosome preparations for cytogenetic analysis were made as follows: colcemid (0.05 g/mL,GibcoBRL, Life Technologies, Grand Island, NY) was added to the tumor cell cultures during the final 20–30 minutes before harvest. The cells were harvested by mitotic shake-off, pelleted by centrifugation, and re-suspended in 0.075 M KCl at room temperature for 30 minutes, Followed by a methanol-acetic acid fixation as described previously [20]. The tumor metaphase chromosomes were subjected to cytogenetic analysis after G-banding.

### M-CGH

M-CGH analysis for all 52 mammary tumors was carried out as described before [21]. In short, equal amounts (1 μg) of test DNA and reference DNA (normal DNA) were differently labeled by nick translation with biotin-16-dUTP (Enzo, Roche) and digoxigenin-11-dUTP (Enzo, Roche), respectively. Probe-DNA hybridizations were performed on normal metaphase slides from rat embryo cell cultures. The probe signals were detected with avidin fluorescein isothiocyanate (FITC) (Vector Laboratories, Burlingame, CA) and rhodamin-digoxigenin (Sigma, Saint Louis, MO). The chromosomes were counterstained with DAPI, dissolved in Vectashield mounting medium (Vector Laboratories). The fluorescence was recorded using the Leica DM RXA microscopy (Wetzlar, Germany) equipped with an incident light fluorescence system, a HBO 100 W vapor mercury lamp, and filter blocks, specific for either fluorochrome, DAPI, or Texas red, in combination with the Leica Q-FISH software developed for microphotography (Cambridge, UK). The Leica CW4000 software package (Cambridge, UK) was used to perform digital image analysis.

### BAC CGH-array

For the purpose of this study, we developed an in-house BAC CGH-array platform, consisting of over 600 BACs that represented 17 chromosome arms. Selection of chromosome segments was made based on the M-CGH results in this study and we included all chromosome segments that showed, even slight, deviation from normal CGH profile. We additionally included chromosome segments with potential implication in tumorigenesis identified in the previous studies on rat cancer models [22-27]. BAC clones were purchased (BACPAC RESOURCES, Oakland, CA), cultured and BAC clone DNA was extracted according to the manufactures instructions. The BAC DNA was then amplified using DOP-PCR (DOP-Primer1; CCGACTCGAGNNNNNNCTAGAA, DOP-Primer2; CCGACTCGAGNNNNNNTAGGAG, DOP-Primer 3; CCGACTCGAGNNNNNNTTCTAG), according to protocol suggested by Fiegler and co-workers [28], PCR products were purified and spotted to glass slides with six individual spots for every BAC on each array.

Based on M-CGH results we selected a total of 28 tumor samples for BAC CGH-array analysis, including 4, 6, and 18 tumors from the SPRD-Cu3, F1 and the backcross genetic backgrounds, respectively (Table 1). Two μg of each tumor and control (normal female liver) DNA was labeled with Cy3 or Cy5, purified and co-hybridized in equal amount on the arrays according to instructions from the manufacture (Invitrogen, Carlsbad, CA). The printing of the array slides, hybridization, wash and scanning of the images were performed at the DNA Microarray Resource Center at SCIBLU (Swegene Centre for Integrative Biology at Lund University, Sweden; http://www.lth.se/sciblu/services/dna_microarrays/). All data was transferred into the BASE 2.9 (BioArray Software Environment) array data analysis software (http://base.onk.lu.se/onk/) and analyzed.

### Oncogenetic tree

Oncogenetic tree analysis was performed as previously described [13,29]. This analysis is a mathematical methodology that uses genetic data to depict the likely order of genetic events occurring during tumorigenesis. We used the Oncotrees software (http://www.ncbi.nlm.nih.gov/CBBresearch/Schaffer/cgh.html) to calculate the weight matrix for all pairs of events and to derive a model tree using the maximum weight branching algorithm, which finds the rooted tree where the sum of the weights of all edges is maximized.

## Results

In the present work, SPRD-Cu3, F1-cross and backcrosses from SPRD-CU3 and WKY strains were used to obtain a set of tumors with different genetic background. Cytogenetic analysis of 10 primary mammary tumor cell cultures derived from SPRD-CU3 female rats, all revealed a diploid genome.

### M-CGH analysis showed recurrent RNO10 and RNO12 and RNO20 gains

**Figure 1:**
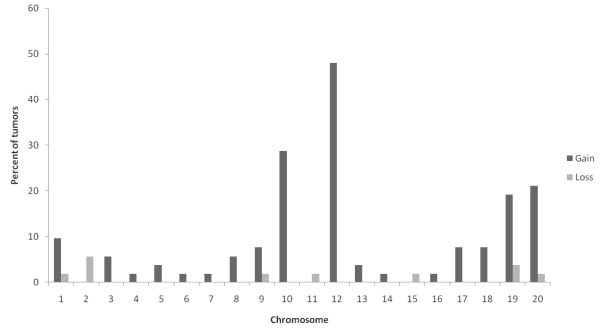
**Summary of the results from M-CGH analysis of 52 DMBA-induced mammary tumors developed in animals from SPRD-CU3 (11 tumors), (SPRD-Cu3xWKY) F1 (6 tumors) and SPRD-Cu3x(SPRD-Cu3xWKY) crosses (35 tumors).** Frequency of chromosomal gains (dark gray bars) and losses (light bars) are presented as percentage of tumors that displayed the aberration.

Fifty-two DMBA-induced mammary solid tumors with different genetic backgrounds were analyzed with M-CGH. For every tumor, 10–15 metaphases were analyzed and an average fluorescence ratio (FR) curve per chromosome was established. Aberrations were recorded for FR curves when these curves displayed values equal to or below 0,85 as losses and equal to or above 1,15 as gains Additional file 1: Table S1. For the majority of the chromosomes, the M-CGH analysis profile revealed FR curves very close to the midline along the entire chromosome, indicating a near normal CGH profile in most of the tumors. However, three chromosomes, RNO10, RNO12 and RNO20, displayed moderate gains in more than 20% of the tumors (Figure 1), particularly in those derived from the inbred SPRD-CU3 and the backcross animals (Additional file 1: Table S1). RNO12 was the most recurrently altered chromosome with gains in 25 (48%) of all tumors. RNO10, and RNO20 gains were less common and were present in 15 (29%) and 11 (21%) of the tumors, respectively (Figure 1). Very few signs of losses were detected among the tumors.

More detailed analysis of RNO10, RNO12 and RNO20 gains revealed that frequency of these genetic changes varied among tumors derived from the three different genetic backgrounds (data not shown). For instance, RNO10 and RNO12 and RNO20 gains were detected in tumors from SPRD-CU3 and the backcross animals, whereas the F1 tumors only displayed gains of RNO10.

### BAC CGH-array analysis confirmed and refined M-CGH findings with a higher resolution

**Figure 2:**
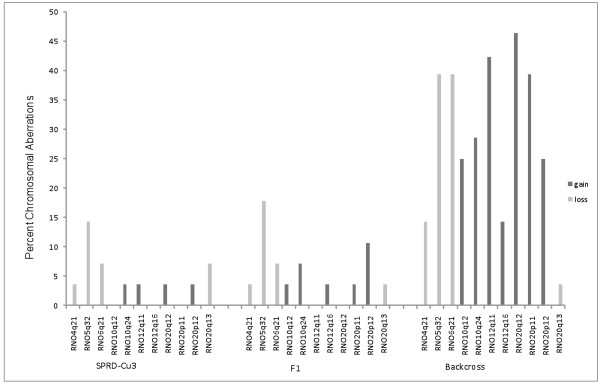
**Incidence of the 11 most recurrent chromosomal gains (dark gray bar) and losses (light bars) as revealed by BAC CGH-array analysis in tumor material.** Results for the three tumor sets derived from different genetic backgrounds are reported separately. As shown, tumors derived from the backcross animals in general displayed more aberrations compared to those derived from SPRD-CU3 and F1 animals. Frequency of each aberration was also found to be different between the tumor sets.

Based on the M-CGH results and availability of the material, we selected a total of 28 tumors, including all the 26 tumors that displayed signs of chromosomal aberrations plus two of those not showing any deviations as controls, for detailed analysis using BAC CGH-array. We developed an in-house BAC CGH-array covering 17 chromosome arms selected based on the results from the M-CGH analysis and the previous studies on rat cancer models [22-27]. Certain chromosome regions containing repetitive DNA were excluded in order to reduce the risk of false results. These included the centromeric and outermost telomeric regions of all chromosomes plus the heterochromatin regions at RNO1q12 ~ q21, RNO3p, RNO9q11 ~ q12, RNO11p, RNO12p, RNO13p, and RNO19p. Since RNO10, 12 and 20 displayed the most recurrent alterations (over 20%) in the M-CGH analysis we chose an average distance between BAC clones of 0.46 to 0.92 Mb for these three chromosomes on the array. High resolution BAC clone coverage was also prepared for chromosome 4, 5 and 6, as earlier studies in our group repeatedly pinpointed to potential significance of aberrations in these chromosomes in tumor development [22,23,27]. For the rest of the chromosomes, BAC clone coverage was less dense with an average distance of 3 Mb. BAC CGH-array analysis of 28 DMBA-induced mammary tumors confirmed and extended the M-CGH analysis results and revealed several recurrent chromosomal gains and losses, each including more than one BAC. The eleven most frequent nonrandom aberrations in the tumor material are summarized in Table 2. RNO20 was the most frequently altered chromosome and was the only chromosome exhibiting both gains (in three regions) and loss (in one region, Table 2). A new finding was frequent chromosomal losses in RNO5q32 (in 20/28 of tumors, 71%), which represented the second most recurrent alteration observed in the present work. Twenty-two tumors showed gains at two different positions of RNO12q making this chromosome the third most altered chromosome in the tumor set. Gains of RNO10 occurred with a frequency of 64% (in 18 cases) followed by loss of RNO6q21 (53%, 15 cases) and RNO4q21 (21%, 6 cases). As expected, the two control tumors with normal karyotype showed a normal chromosome content in the BAC CGH-array analysis. Results from the BAC CGH-array analysis for tumors derived from different genetic backgrounds are summarized in Figure 2.

**Table 2:** The most recurrent chromosomal aberrations detected by BAC CGH-array in a panel of 28 SPRD-CU3 DMBA-induced mammary tumors

**RNO**	**Position (Mb) in the rat genome**	**Rat position**	**Homologous position in human**	**Frequency**
Loss				
5q	~108	5q32	9p21	20 (71%)
6q	~60-66	6q21	14q12, 7q22	15 (53%)
4q	~38-43	4q21	7p21, 7p31	6 (21%)
20q	~49-telomere	20q13	6q21	4 (41%)
Gain				
12q	~15	12q11	7p22	15 (53%)
20q	~44	20q12	6q21	14 (50%)
20p	~15	20p11	10q21	12 (43%)
20p	~3	20p12	UN	11 (39%)
10q	~54	10q24	17p13	11 (39%)
10q	~13	10q12	16p13	8 (28%)
12q	~44	12q16	12q24	5 (18%)

Based on Human 35 and Rat RGSC v3.4 genome build.

### Analysis of BAC CGH-array results by oncogenetic tree identified early and late events

**Figure 3:**
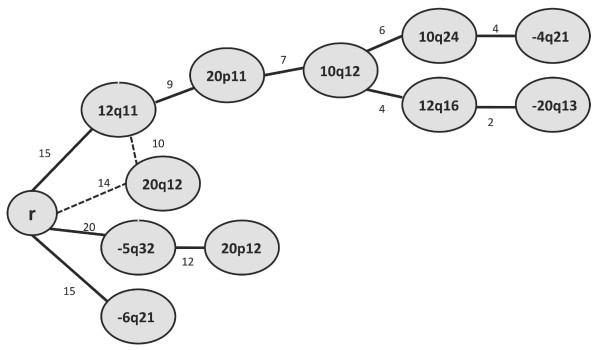
**Order of genetic events predicted by maximum weight branching tree.** The most frequent events identified by BAC CGH-array analysis were included in this analysis. “r” represents the root, *i.e.* the normal cell from which the oncotree (the pathogenic road) started. Numbers illustrated along the paths represent the number of tumors contributing to the development path that led to each node. The dashed lines represent the two possible alternative paths.

We performed an oncogenetic tree analysis on the BAC CGH-array data from analysis of 28 tumors (Figure 3). The analysis suggested gain of RNO12q11, and loss of RNO5q32 and RNO6q21 as the earliest events in mammary tumorigenesis in this material. In addition, gain of RNO20q12 was placed as a very early deviation that could occur either as a second event on the RNO12q11 path or as an independent initial single event. From the initial event of RNO12q11 gain, a series of later genetic events were branched, among which loss of RNO4q21 and RNO20q13 represented the latest deviations in this path. From the initial event conferring loss of 5q32, gain of 20p12 followed as a single node. Loss of RNO6q21 occurred as an isolated event branched independently from the root (Figure 3).

## Discussion

The exact molecular mechanisms responsible for the onset and progression of breast cancer are still poorly understood. Unlike the majorities of solid tumors, breast cancer is usually associated with multiple small-scale genetic alterations, including minor amplifications and deletions in specific chromosomal regions. In the present work, we combined classic cytogenetic analysis (G-banding) with advanced molecular methodologies (M-CGH and BAC CGH-array analysis), as well as mathematical algorithms to find and define specific genetic changes and events leading to tumor development in DMBA-induced mammary tumors in a well-defined rat model for the disease.

G-banding analysis of 10 tumors derived from inbred SPRD-CU3 animals revealed a diploid karyotype in all. This result is in concordance with previously reported cytogenetic data for synthetic chemically (DMBA or NMU)-induced rat mammary tumors [30-32], but contrasts with the reports from estradiol-induced ACI mammary tumors [32,33]. M-CGH analysis of 52 tumors from three different genetic backgrounds partly confirmed this observation and showed that most of the chromosomes in the majority of tumors had profile ratios quite close to the midline, indicating a close to normal karyotype. However, recurrent segmental gains were detected in three chromosomes; RNO10, RNO12 and RNO20 (Figure 1). This type of segmental gains cannot be detected by G-banding due to the limitation of this technique in detecting milder aberrations in sub group of the cells. When we studied the three tumor sets derived from the different genetic backgrounds separately (data not shown), frequency of these genetic changes appeared to vary among the tumor groups. For instance, gain of RNO12 was the most common alteration followed by RNO10 and RNO20 gains in tumor groups derived from SPRD-CU3 and the backcross animals, whereas the F1 tumors exclusively displayed gains of RNO10. The limited number of F1 tumors (six compared to 11 SPRD-Cu3 and 35 backcross tumors) might have affected this result, however, our data suggest that the nature of genetic alterations might be influenced by the genotype of the animals. In general, not many chromosome losses were detected in the tumor panel using this technique. This was most likely due to less sensitivity of M-CGH technique in detecting segmental chromosomal losses compared to chromosomal gains [34]. Taken together, M-CGH analysis suggested a nonrandom pattern of chromosome segment gains of RNO10, RNO12 and RNO20 in the tumor material and also implied that frequency of these aberrations varied in the tumors developed in animals with different genetic backgrounds.

To obtain a higher resolution of the genetic events, we then performed BAC CGH-array analysis on a panel of 28 tumors selected based on the M-CGH data, including all 26 tumors that displayed signs of chromosomal changes and two tumors showing a normal M-CGH profile. For the purpose of this analysis, we developed an in-house BAC CGH-array platform designed to provide a high density for chromosomal regions of interest. The analysis confirmed and extended the M-CGH data and refined the affected chromosome regions to a resolution of about 150–300 Kb (*i.e.* the average size of the BAC clones). We subsequently derived an oncogenetic tree model based on the BAC CGH-array data, which suggested four of the observed recurrent chromosomal changes as possible initial events in mammary tumor development in this model.

The most recurrent genetic changes were as follow:

RNO5: Loss of RNO5q32 was the single most recurrent segmental alteration detected in the BAC CGH-array analysis of DMBA-induced mammary tumors (in 20 tumors, 71%). RNO5q32 loss was not detected by M-CGH due to less sensitivity of this technique in detection of segmental losses [34]. This event was moreover placed as one of the initial events in the oncogenetic tree model, suggesting an important role for the gene(s) located at this chromosomal segment in the development of DMBA-induced mammary tumors. RNO5q32 is homologous to human chromosome 9p21 that among a number of important tumor suppressor genes, harbors the *CDKN2A/2B* locus with its deletion frequently reported in different cancer types, including breast cancer [35]. This finding was more intriguing, since M-CGH study of estradiol-induced mammary tumors in ACI rats similarly reported loss of RNO5 as the most frequently observed somatic abnormalities that usually occurred together with RNO20 losses [33]. Our preliminary BAC CGH-array analyses of a set of ACI estradiol-induced mammary tumors confirmed and extended this finding (unpublished data). We thus conclude that deletion at this chromosome segment might represent a common signature of experimental mammary tumors (irrespective of the genetic background and the inducing agent) and thus may represent an important genetic mechanism in breast cancer development.

Remarkably, RNO5 is reported to carry mammary cancer susceptibility QTLs (*Mcs5Mcstm1Emca1*, and *Emca8*) in different rat mammary tumor models that control multiplicity, incidence and/or latency of tumors [7,36-38]. Some of these QTLs are shared between strains and thus appear in different crosses, while others are specific to one cross or limited to a few crosses [7]. In general QTLs cover large chromosome intervals and therefore identification of the gene(s) responsible for the phenotype is difficult. Recurrent RNO5q32 losses detected in this model might explain some of the reported QTLs and as the genetic changes identified in this work are at very small scale, identification of the target gene(s) might be more feasible.

RNO20: Four separate recurrent chromosomal aberrations were detected in RNO20, including three regions of recurrent gains (RNO20p11, 20p12 and 20q12) and one region of recurrent loss (RNO20q13). Together, these regions make RNO20 the most frequently affected chromosome in this investigation. In the oncogenic tree model, RNO20p11 and RNO20p12 gains were placed close to the root, but as secondary events after RNO12q11 gain and RNO5q32 loss, respectively (Figure 3). The third gained chromosomal segment at cytogenetic band RNO20q12 was placed as an alternative path prior to the RNO12q11 gain and thus might represent an early and important event in the development of DMBA-induced mammary tumors. Loss of RNO20q13 was placed as one of the latest events in the RNO12q11 path and thus might be of less importance in this model. Interestingly, M-CGH analysis of ACI estradiol-induced mammary tumors [33] showed that recurrent RNO20 losses were correlated to RNO5 deletions. In our study, we did not reach a similar conclusion; instead we found evidence for correlation of RNO20p12 gain and RNO5q32 loss in a sizable proportion of tumors. We suggest that RNO20 gains (RNO20p11, 20p12 and 20q12) might be more specific to the DMBA-induced mammary tumor model. Since these aberrations were present in a substantial proportion of experimental mammary tumors, we suggest that RNO20 gains might represent another key group of genetic changes in mammary cancer development. Gains of the homologous human chromosome regions (6p21 and 6q21) might be implicated in breast cancer development, at least in a group of patients.

RNO6q21 loss and RNO12q11 gain were the next most frequently observed chromosome aberrations in this material, each observed in 15 of the tumors (53%). In the oncogenic tree model, RNO6q21 loss was branched independently from the root, defining a subgroup of tumors independent from those with RNO5q32 deletion or RNO12q21 gain (Figure 3). Our finding was more intriguing, since M-CGH analysis of ACI estradiol-induced rat mammary tumors [33], reported similar finding of a recurrent aberration involving proximal gain and distal loss of RNO6 that never occurred in combination with RNO5 loss. Our result thus suggests that the genetic pathways (at least those involving genes located at RNO5q32 and RNO6q21) related to tumor formation in DMBA-induced rat mammary tumor are comparable to those in the ACI estradiol-induced rat model. RNO6q21 is homologous to parts of human chromosome 14q12 and 7q22. The human chromosome segment 7q22 contains an evolutionary breakpoint between the rat and human lineage. Deletion in this region is frequently reported as a primary breakpoint in cancer and has been suggested to be due to the fragility of this particular segment [39].

RNO12q11 gain was found as the third initial event branching from the root in the oncogenic tree model (Figure 3). This event was additionally placed as secondary event after an alternative path initiated by RNO20q12 gain in the oncogenic tree (Figure 3). There were two overlapping BACs in this chromosome segment in BAC CGH-arrays, both showing gains in a substantial subset of tumors. The gene *Mafk* (v-maf musculoaponeurotic fibrosarcoma oncogene family, protein K) is located in both of these clones suggesting that the *Mafk* gene might be the potential target. *Mafk* is known to be involved in transcription regulation and its implication has already been suggested in pancreatic cancer [40].

 Finally, gain of RNO10 (at 10q12 and 10q24) appeared along the pathogenic road initiated by gain of RNO12q11 in the oncogenic tree (Figure 3). In all crosses, RNO10 gains were found at cytogenetic bands RNO10q12 and RNO10q24, each segment including several BACs.

## Conclusions

Taken together, M-CGH analysis suggested RNO10, 12 and 20 gains as the most recurrent chromosomal changes among tumors. BAC CGH-array analysis confirmed this finding and extended it by refining the affected chromosome segments to a resolution of about 150–300 Kb. However, the identified genetic background-specific chromosomal gains by M-CGH analysis were not confirmed by BAC CGH-array data. This could be due to the limited number of tumors derived from SPRD-Cu3 and F1 animals in the tumor panel that was used for the BAC CGH-array analysis. BAC CGH-array analysis could additionally identify recurrent chromosome segment losses, which could not be detected by M-CGH. This can be explained by substantially higher resolution and sensitivity of this technique, when compared to M-CGH. It is important to note that for BAC CGH-array analysis, we selected a panel of 28 tumors based on M-CGH results, including all the 26 tumors with signs of chromosomal aberrations and two control tumors with normal chromosome profiles. Although the two control tumors did not show any chromosome aberrations in the BAC CGH-array analysis, there is still a possibility that among the remaining 24 tumors, there existed tumor(s) with minor aberrations (not detectable by M-CGH and thus excluded from the analysis) that might have been overlooked.

In summary, we identified recurrent sub-microscopic chromosome gains and losses in diploid SPRD-CU3 DMBA-induced mammary tumors. Using oncogenic tree analysis, we classified these aberrations to early and late events. Some of the chromosome anomalies, including gains in three independent minimal segments of RNO20 (RNO20p11. 20p12 and 20q12) and in a small segment of RNO 12q11, appeared to be more specific to this tumor model, since they have not been reported in other mammary tumor models [33]. Since these aberrations were observed in a substantial number of tumors and also the oncogenic tree analysis identified them as early events, we suggest that they may represent a key group of genetic changes with potential implication in breast cancer development, at least in a subgroup of patients. RNO12q11 was represented in the array by two overlapping BACs, both harboring the *Mafk* gene, whose implication has earlier been reported in pancreatic cancer [40].

Another group of chromosome anomalies, namely RNO5q32 and RNO6q21 losses, are similar to those found in ACI estradiol-induced mammary tumors [33]. Since these chromosome aberrations are strain- and inducing agent-independent and again were placed as early events in the oncogenic tree, they seem critical to mammary tumor development.

Identifying the genes underlying chromosome changes found in the present work should help in understanding biological mechanisms involved in mammary development. Genetic changes identified in the present work are at very small scale and may thus make identification of the potential target gene(s) more feasible. Such studies should yield both genetic and biological information useful to understanding of human breast cancer.

## Abbreviations

ACI, AxC-Irish rat strain; BAC, Bacterial artificial chromosome; CGH, Comparative genome hybridization; CDKN2A/2B, Cyklin-dependent kinase 2A and 2B; DMBA, 7,12-dimethylbenz[a]anthrazene; Emca, Estogen-induced mammary cancer; Mafk, V-maf musculoaponeurotic fibrosarcoma oncogene family, protein K; Mb, Megabases; M-CGH, Metaphase comparative genome hybridization; Mcs, Mammary cancer susceptibility; Mcstm, Mammary cancer susceptibility, tumor multiplicity; NMU, (N-methyl-nitrosurea); QTL, Quantitative trait locus; RNO, Rat chromosme; SPRD-Cu3, A curly mutant of Srague-Dawley rats; WKY, Wistar Kyoto rats.

## Competing interests

The author(s) declare that they have no competing interests.

## Authors' contributions

ES carried out keryotyping, CGH and BAC CGH-array experiments, participated in the design of the study, performed the analysis and helped to draft the manuscript. SK participated in the design and production of BAC arrays. KP participated in the analysis of BAC CGH-array data. SN performed the statistical analysis. CS performed the animal crosses and provided the tumor material as well as helped to draft the manuscript. AB conceived the study, performed the study design and coordination and drafted the manuscript. All authors read and approved the final manuscript.

## Pre-publication history

The pre-publication history for this paper can be accessed here:

http://www.biomedcentral.com/1471-2407/12/352/prepub

## Supplementary Material

Additional file 1 **Table S1.** M-CGH analysis results in all 52 tumors. Summary of the results is presented in Figure 1. Click here for file
