# Supplementary material for: BAC CGH-array identified specific small-scale genomic imbalances in diploid DMBA-induced rat mammary tumors
Source: BMC Cancer. 2012 Aug 15;12:352. doi: 10.1186/1471-2407-12-352 (PMC3488521; doi:10.1186/1471-2407-12-352)
Supplement: Additional file 1 — Table S1. M-CGH analysis results in all 52 tumors. Summary of the results is presented in Figure 1. [file 1471-2407-12-352-S1.doc]

**Supplementary table.** M-CGH analysis results in all 52 tumors. Summary of the results is presented in Figure 1.

| **Tumor** | **Chromosome** | | | | | | | | | | | | | | | | | | | | |
| --- | --- | --- | --- | --- | --- | --- | --- | --- | --- | --- | --- | --- | --- | --- | --- | --- | --- | --- | --- | --- | --- |
|  | 1 | 2 | 3 | 4 | 5 | 6 | 7 | 8 | 9 | 10 | 11 | 12 | 13 | 14 | 15 | 16 | 17 | 18 | 19 | 20 | X |
| **SPRD-Cu3** |  |  |  |  |  |  |  |  |  |  |  |  |  |  |  |  |  |  |  |  |  |
| 1 |  |  |  |  |  |  |  |  |  | + |  | + |  |  |  |  |  |  |  |  |  |
| 2 | + |  | + |  | + |  | + |  | + | + |  | + |  |  |  |  |  |  | + |  |  |
| 3 |  |  |  |  |  |  |  |  |  | + |  | + |  |  |  |  |  |  |  | + |  |
| 4 |  |  |  |  |  |  |  |  |  |  |  | + |  |  |  |  |  |  |  |  |  |
| 5 |  |  |  |  |  |  |  |  |  |  |  |  |  |  |  |  |  |  | − |  |  |
| 6 |  |  |  |  |  |  |  |  |  |  |  | + |  |  |  |  |  |  | − |  |  |
| 7 | + |  | + |  |  |  |  |  | + |  |  | + |  |  |  |  |  |  | + |  |  |
| 8 |  |  |  |  |  |  |  | + |  | + |  |  |  |  |  |  |  |  |  |  |  |
| 9 |  |  |  |  |  |  |  |  |  |  |  |  |  |  |  |  |  |  |  |  |  |
| 10 |  |  |  |  |  |  |  |  |  |  |  |  |  |  |  |  |  |  |  |  |  |
| 11 | + |  |  |  |  |  |  |  |  |  |  |  |  |  |  |  |  |  |  |  |  |
| (**SPRD-Cu3xWKY)F1** |  |  |  |  |  |  |  |  |  |  |  |  |  |  |  |  |  |  |  |  |  |
| 12 |  |  |  |  |  |  |  |  |  |  |  |  |  |  |  |  |  |  |  |  |  |
| 13 |  |  |  |  |  |  |  |  |  |  |  |  |  |  |  |  |  |  |  |  |  |
| 14 |  |  |  |  |  |  |  |  |  |  |  |  |  |  |  |  |  |  |  |  |  |
| 15 |  |  |  |  |  |  |  |  |  | + |  |  |  |  |  |  |  |  |  |  |  |
| 16 |  |  |  |  |  |  |  |  |  | + |  |  |  |  |  |  |  |  |  |  |  |
| 17 |  |  |  |  |  |  |  |  |  |  |  |  |  |  |  |  |  |  |  |  |  |
| (**SPRD-Cu3xWKY)N1** |  |  |  |  |  |  |  |  |  |  |  |  |  |  |  |  |  |  |  |  |  |
| 18 |  |  |  |  |  |  |  |  | - |  |  | + |  |  |  |  |  |  |  |  |  |
| 19 |  |  |  |  |  |  |  |  |  | + |  |  | + | + |  |  |  |  |  | + |  |
| 20 |  |  |  |  |  |  |  |  |  | + |  | + |  |  |  |  |  |  |  |  |  |
| 21 |  |  |  |  |  |  |  |  |  |  |  | + |  |  |  |  |  |  |  |  |  |
| 22 |  |  |  |  |  |  |  |  |  | + |  | + |  |  |  |  |  |  |  |  |  |
| 23 |  | − |  |  |  |  |  |  |  | + | − | + |  |  |  |  |  |  |  |  |  |
| 24 |  |  |  |  |  |  |  |  |  |  |  | + |  |  |  |  |  |  |  |  |  |
| 25 |  |  |  |  |  |  |  |  |  |  |  |  |  |  |  |  |  |  |  | + |  |
| 26 |  |  |  |  |  |  |  |  |  |  |  | + |  |  |  |  |  | + |  | + |  |
| 27 |  | − |  |  |  |  |  |  |  |  |  | + |  |  | − |  |  |  |  |  |  |
| 28 |  |  |  |  |  |  |  |  | + | + |  | + |  |  |  | + | + | + | + | + |  |
| 29 |  |  |  |  |  |  |  |  |  |  |  | + |  |  |  |  |  |  |  |  |  |
| 30 |  |  |  |  |  |  |  |  |  |  |  | + |  |  |  |  |  |  |  |  |  |
| 31 |  |  |  |  |  |  |  | + |  | + |  | + |  |  |  |  |  |  | + | + |  |
| 32 | + |  |  | + |  | + |  | + | + | + |  | + | + |  |  |  | + | + | + | + |  |
| 33 |  | − |  |  |  |  |  |  |  |  |  | + |  |  |  |  | + |  | + |  |  |
| 34 |  |  |  |  |  |  |  |  |  |  |  | + |  |  |  |  |  |  |  | + |  |
| 35 |  |  |  |  |  |  |  |  |  |  |  | + |  |  |  |  |  |  |  |  |  |
| 36 | + |  |  |  |  |  |  |  |  | + |  | + |  |  |  |  | + |  | + | + |  |
| 37 |  |  |  |  |  |  |  |  |  | + |  | + |  |  |  |  |  | + | + | + |  |
| 38 |  |  |  |  |  |  |  |  |  |  |  |  |  |  |  |  |  |  |  |  |  |
| 39 |  |  |  |  |  |  |  |  |  |  |  |  |  |  |  |  |  |  |  |  |  |
| 40 |  |  |  |  |  |  |  |  |  |  |  |  |  |  |  |  |  |  |  |  |  |
| 41 |  |  |  |  | + |  |  |  |  |  |  |  |  |  |  |  |  |  |  |  |  |
| 42 |  |  |  |  |  |  |  |  |  |  |  |  |  |  |  |  |  |  |  |  |  |
| 43 |  |  |  |  |  |  |  |  |  |  |  |  |  |  |  |  |  |  |  |  |  |
| 44 |  |  |  |  |  |  |  |  |  |  |  |  |  |  |  |  |  |  |  |  |  |
| 45 |  |  |  |  |  |  |  |  |  |  |  |  |  |  |  |  |  |  |  | − |  |
| 46 |  |  |  |  |  |  |  |  |  |  |  |  |  |  |  |  |  |  |  |  |  |
| 47 |  |  |  |  |  |  |  |  |  |  |  |  |  |  |  |  |  |  |  |  |  |
| 48 |  |  |  |  |  |  |  |  |  |  |  |  |  |  |  |  |  |  |  |  |  |
| 49 |  |  |  |  |  |  |  |  |  |  |  |  |  |  |  |  |  |  |  |  |  |
| 50 |  |  |  |  |  |  |  |  |  |  |  |  |  |  |  |  |  |  |  |  |  |
| 51 |  |  |  |  |  |  |  |  |  |  |  | + |  |  |  |  |  |  | + | + |  |
| 52 | − |  |  |  |  |  |  |  |  |  |  |  |  |  |  |  |  |  | + |  |  |
